# The history of peritoneal dialysis in China: past, present and future trends

**DOI:** 10.1080/0886022X.2021.2011316

**Published:** 2021-12-04

**Authors:** Shu-Hong Bi, Weiyu Chen, Jimmy S. Wu, Tao Wang, Suhail Ahmad

**Affiliations:** a Department of Nephrology, Peking University Third Hospital, Beijing, China; b Department of Cardiology, Cardiovascular Hospital Affiliated to Xiamen University, Xiamen, China; c School of Medicine, University of Washington, Seattle, Washington, USA

**Keywords:** Peritoneal dialysis, history, China, registration

## Abstract

Peritoneal dialysis (PD) was introduced in China more than 60 years ago and has grown continuously since then. Now China leads the first of the world in number of patients on PD. In this manuscript a brief review of the history of peritoneal dialysis in China is presented; this includes a description of pioneers and their important contributions, discussion of peritoneal dialysate, the technique of the use of Tenckhoff catheter, the use of continuous ambulatory peritoneal dialysis (CAPD) and dialysis registration. Current ongoing PD research activities among Chinese PD academicians are also discussed. Finally, we present four areas of future focus: 1) the promotion of PD in rural areas where PD use is still very limited due to the lack of PD awareness and education; 2) PD quality management and continuous quality improvement (CQI) program particularly focusing on PD adequacy and patient rehabilitation; 3) development and enforcement of national standards on PD management; 4) multi-center studies to compare the benefits of PD and hemodialysis (HD) that should include survival, rehabilitation and cost-effectiveness.

In the history of medicine in general and Nephrology in particular the pioneering innovative use of peritoneal dialysis (PD) is a ground-breaking development. Currently, in China PD is an important form of renal replacement therapy (RRT), and it is important to document the history of PD in China that is briefly presented in this manuscript.

Similar to other parts of the world, in China interest in PD as a RRT technique has been evident from early 1950s. As early as in 1950s the use of PD had been explored by Chinese researchers; however the clinical use of PD did not begin until 1962. Some of these early important works have been briefly discussed in the following sections.

The earliest account of clinical use of PD is from Xiangya Hospital of Central South University in Hunan Province in 1954 by Dr.Wu Hanwen (伍汉文). He and his colleagues successfully used intermittent PD (IPD) to treat a patient with acute renal failure (ARF) caused by mercury poisoning. Dr.Wu Hanwen used a urethral catheter with multiple lateral holes as the dialysis catheter, and glucose saline solution as dialysate [1].

Later in the 1960s and 1970s PD was used in several hospitals in Guangzhou, Tianjin and Beijing. Professor Ye Rengao (叶任高, Figure 1), a nephrologist at The First Affiliated Hospital of Sun Yat-sen University in Guangzhou, and Professor Zhao Zhigang (赵之纲), a physician at the First Center Hospital of Tianjin, were the early pioneers to use PD to treat patients with acute and chronic renal failure (CRF) in 1962 and 1964 respectively [2,3]. These two are generally credited as being the first to utilize PD as a clinical procedure in China.

**Figure 1.**
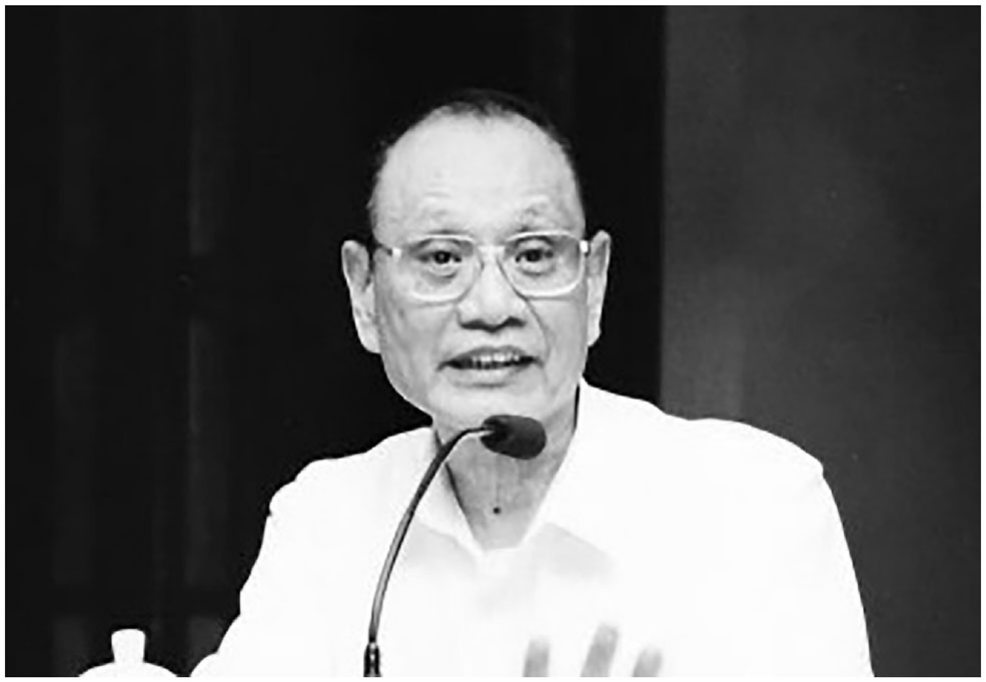
Dr. Ye Rengao (叶任高), who was the first to use peritoneal dialysis to treat patients with renal failure in 1962 in China.

In 1975 Professor Zhao and colleagues reported their experience of PD from 1964 [3]. They described that after several days of PD treatment in 5 patients with ARF, renal function in 4 patients completely recovered and 5 of 8 patients with CRF had marked improvement in their clinical symptoms on PD.

In the same year Professor Gu Fangliu (顾方六), a urologist in Peking University First Hospital, reported their successful experience of PD use. PD was used in 11 patients with ARF; one died of sepsis, and 10 patients survived with complete recovery of their renal function [4].

In 1983 Professor Ye reported their experience in patients with acute and chronic renal failure using PD [5,6]. They described the use of PD in 48 patients with ARF starting in 1962. Of the 48 patients, 15 died of sepsis, 20 patients had complete recovery after about 10 days of PD treatment [5]. They also discussed their experience in 41 patients with CRF from 1964 to 1980. With PD uremia symptoms were alleviated and patients survived; the longest surviving patient lived for 302 days [6].

These early experience highlighted the safety and efficacy of PD as a viable RRT increasing confidence and secure feeling in Chinese nephrologists in this technique.

## PD catheter and implantation techniques

For short-term PD in the treatment of ARF, a medical silicone-rubber catheter with small lateral wall holes was used [7]. As discussed above a modified urethral catheter was also used as a PD catheter with added 8-9 small holes on the side wall of the catheter [3].

For patients with CRF long-term PD was needed. Initially for long-term use in CRF patients a silicone-rubber catheter was used, however complications such as migration of the tip, fluid leak and peritonitis were quite common limiting its use [7,8]. In 1979 the introduction of Tenckhoff catheter in China significantly reduced the occurrence of catheter-related complications and promoted the use of PD in CRF [7]. Both percutaneous insertion technique and surgical placement of catheter were used.

Percutaneous catheter placement using Seldinger technique with a trocar was frequently used in emergency dialysis or for short-term PD [3].

Open surgical approach was usually used for long-term PD. First the end of the PD catheter was placed in the cystorectal fossa. The Dacron cuff at the proximal end of PD catheter was then buried between the subcutaneous fat and the anterior sheath of rectus abdominis, and a 10-12 cm long tunnel was made under the adipose layer of the abdominal wall to bury the subcutaneous tunnel part of the catheter. The PD catheter would then be pulled out from the tunnel exit site leaving the distal Dacron cuff under the skin [7]. The surgical technique remains the most widely used method in clinical practice in China [9].

## The use of Tenckhoff catheter made long-term PD possible

In the early 1960s and 1970s in China, PD had been recognized as an effective RRT to treat acute and chronic renal failure. However peritonitis remained a significant complication, limiting its use. In 1979 the availability and use of Tenckhoff catheter greatly reduced peritonitis and made long-term PD possible.

Tenckhoff catheter is a kind of silicone tube [Silastoic] designed by Professor Tenckhoff of University of Washington, Seattle, USA in early to mid 1960s [10]. The silicone rubber catheter has two polyester Dacron cuffs, one under the skin and the other external to fascia lining the parietal peritoneal membrane. In 10-14 days after the implantation the connective tissue grows into the Dacron cuff effectively providing a seal around the catheter thus preventing infection and leakage of fluid. And the curved design of the sub-cutaneous tunnel also reduces the risk of catheter tip migration. We believe that this critical innovation was a milestone in the history of PD, making wider use of PD possible [10,11].

Professor Ye in July 1979 introduced Tenckhoff catheter in China [12]. The original Tenckhoff catheter to this day remains the most common catheter used for long-term PD [9].

## Dialysate

In 1962 Professor Ye began to use self-made bottled dialysate for PD. The basic formula of dialysate was 5.67 g sodium chloride, 0.26 g calcium chloride, 0.15 g magnesium chloride, 3.92 g sodium lactate and 15 g glucose per liter. If there was no hyperkalemia in these patients, 0.3 g/L potassium chloride was also occasionally used. Glucose 40 g/L was added if more ultrafiltration was needed [5].

Currently dialysate in bags prepared commercially by Baxter Company with lactate as buffer is most commonly used in China. The composition of the dialysate is 5.38 g sodium chloride, 0.26 g calcium chloride, 0.05 g magnesium chloride, 4.48 g sodium lactate per liter. These are available in three concentrations of glucose: 15, 25 and 42.5 g per liter [9].

Two special dialysates, icodextrin dialysate and amino acid containing dialysate are used outside China. These dialysates are not available in China thus are not used. [9,13,14].

Dialysate is also being produced by domestic Chinese companies but its use is limited. In 2013 a multi-center, prospective, randomized, parallel-controlled clinical trial compared the domestic dialysate (Changfu dialysate) and dialysate from Baxter Company. The results showed that the dialysis adequacy and ultrafiltration were very similar in patients using either dialysate. However peritonitis appeared to be more prevalent in the group using domestic dialysate [15]. As a result of this study a series of technical improvements in domestic dialysate production and quality control have been implemented. It is estimated that at the present time, domestic dialysate accounts for about 20% of the total use (unpublished data).

## Development of PD access: tubing and connections

Initially, the Y-rubber connecting lines were used for PD. After each use, the lines were sterilized and reused. However, with repeated high-pressure disinfection, the inner wall of the rubber connecting lines would get damaged, permitting the growth of bacteria thus increasing the risk of peritonitis [16]. From 1983 only single use connecting lines have been used with a significant decrease in peritonitis [16].

In China from 1978 to 1982, bottled dialysate was used; however it required preparation in hospitals which was not suitable for patients who were dialyzed at home [17].

In 1973 the Baxter Company developed peritoneal dialysate in plastic bags, which had the advantage of being more convenient and portable. At the end of 1978 the use of bagged dialysate was approved by the FDA in USA, leading to a switch from IPD to continuous ambulatory peritoneal dialysis (CAPD) [11].

From 1979 to 1992 domestic PD dialysate in 1 liter bags with single infusion line was used in China. The domestic PD dialysate in bags was connected with a single connecting tube (spiking system); the technique did not involve an initial step of flushing the line, consequently the risk of peritonitis was higher [16,17]. Furthermore there is a need to roll up the empty dialysis bag which was inconvenient and often increased the tension on the PD catheter. Due to these factors only a few patients used the domestic bag dialysate system [16–18].

It was not until October 1992 that the PD O-type tubing (O-set) provided by Baxter Company became available in China and started to be used clinically. This commercial set-up consisted of a bag dialysate, the O-tube and a transitional short tube. The procedure involves the flushing of the connecting lines with dialysate, disconnecting the empty bag and the filling of the small connecting tube with disinfectant. This technique made PD more convenient and lowered the risk of peritonitis making home PD possible [16–18].

However if the procedure was not followed properly, some disinfectant could be drained into the peritoneal cavity causing chemical peritonitis. In 1994 Baxter Company introduced the dual tubing with integrated design (twin-bag system), making it less likely to cause disinfectant exposure. This reduced the incidence of chemical peritonitis [18].

The use of Tenckhoff catheter and the development of PD access (Y-set, O-set and twin-bag system) effectively reduced peritonitis, leading to a significant increase in PD population and home dialysis in China [16–18].

## Continuous ambulatory peritoneal dialysis (CAPD): an important development

The concept of CAPD was proposed by Popovich and Moncrief in 1976, initially called ‘equilibrium dialysis.’ Later the technique was renamed CAPD [19,20]. In September 1977 Dr. Oreopoulos (Dimitrios G. Oreopoulos, Canada) reported their experience with CAPD in clinical practice that formally established the model of CAPD in Canada [11]. The continuous dialysis was found to be better for patients in maintaining the uremic control than the usual technique of IPD used until that time.

**Figure 2.**
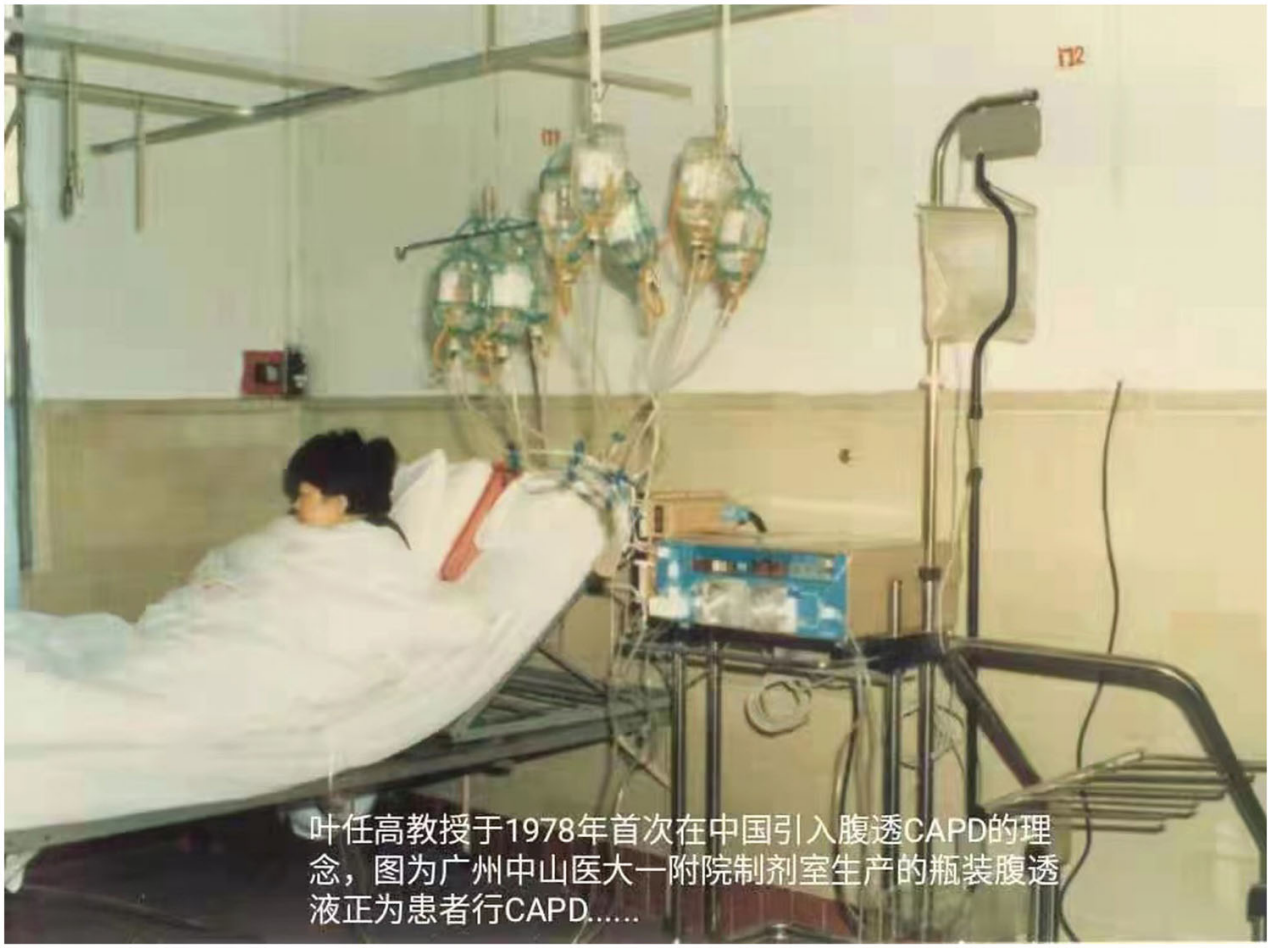
In 1978 Dr. Ye Rengao (叶任高) first introduced the concept and technology of continuous ambulatory peritoneal dialysis (CAPD) into China. This picture showed a patient with renal failure was on CAPD using dialysis solutions in glass containers.

The development of PD in China closely followed the development of the global practice and use of PD. In 1978 Professor Ye first introduced the concept and technology of CAPD into China. In 1979 he led the application of CAPD in patients with renal failure in The First Affiliated Hospital of Sun Yat-sen University, Guangzhou [21, Figure 2]. Meanwhile he and his team popularized the concept of CAPD to the whole country through the advanced-training course for the Chinese senior nephrologists. His efforts resulted into the successful establishment of PD centers in several large hospitals in Guangzhou, Shanghai, Beijing and Nanjing [22,23]. By May 1982 there were 23 PD centers in China treating 256 patients with CAPD [22].

CAPD remains the main modality of PD in China. According to the national registration data in 1999 [24], 86% of PD patients were treated with CAPD. Data from the Chinese National Renal Data System (CNRDS) in 2017 [25] also showed that CAPD treatment remained the main modality of PD, 88.8% patients being treated with CAPD.

## Automated peritoneal dialysis (APD)

In 1962 Norman Lasker first thought of and developed the chronic PD cycler [26]. This cycler used a four-pronged connector joined to four 2-liter glass containers of dialysate. Through this set of tubings, 2 liters of fluid by gravity streamed into a bag that was heated in a heater; the warm fluid from the bag was drained into the peritoneal cavity by gravity. After a determined ‘dwell time’ the used dialysate was drained out into a bag and measured [11]. In 1965 Tenckhoff developed and used his unique cycler at University of Washington in Seattle. This used two 40 liter bottles and one 3 liter bottle, the latter was raised at a height, desired volume of dialysate (usually 2-3 liters) from the 3 liter bottle filled the peritoneal cavity by gravity. The fluid was left in the peritoneal cavity for the desired dwell time after that it was drained to the bottom 40 liter bottle. The cycling of inflow, dwell and drain times were automated and controlled by opening and closing of clamps. The completely closed system and sterile technique was used for more than 15 years at University of Washington without any infection caused by the cycler. The above two cyclers are the predecessor of the current APD automatic machine.

In 1981 Professor Ye first reported their experience of APD with domestic semi-automatic and automatic dialysis machines (XFT-5 and FT-T-73) [6]. After that Shanghai Renji Hospital and Peking University First Hospital started using APD in their patients [27,28]. However due to many factors, including reimbursement issues (Cycler is not paid for requiring patients to buy it using their own money) APD has not been widely used in China. According to the national registration data in 2017, APD accounted for only 1.3% of all PD in China [25].

## Registration of peritoneal dialysis patients

In 1996 Professor Qian Jiaqi (钱家麒) took the lead in carrying out PD registration in China at Shanghai Renji Hospital. He also established the first dialysis electronic registration system in Shanghai [29].

**Figure 3.**
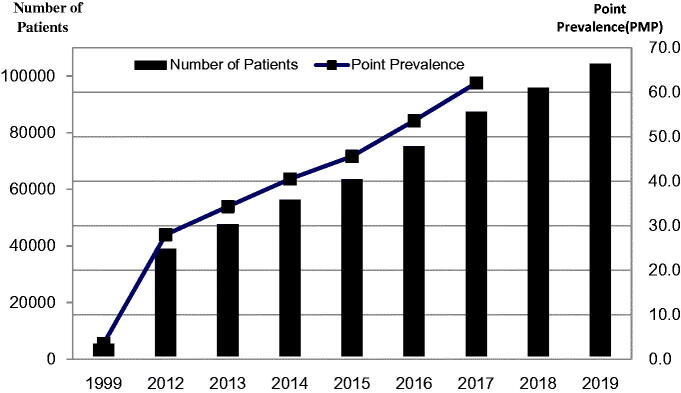
Numbers of peritoneal dialysis patients and point prevalence rates, in patients per million (PMP) from 1999 through 2019.

The national end stage renal disease (ESRD) registration in China was first launched in 1999 [24]. At that time 41,755 ESRD patients were receiving dialysis. PD accounted for 10.5% of all dialysis patients (Figure 3); the point prevalence of PD was 3.5 patients per million population (PMP) [24,30]. In the same year, 2051 new patients started peritoneal dialysis [24, 30].

Since 1999 a gradual and consistent increase in the number of dialysis patients has been seen. A national survey reported 65,074 patients receiving dialysis (HD and PD) at the end of 2007. This number increased to 102,863 by the end of 2008. The point prevalence of dialysis patients in 2007 and 2008 were 51.7 PMP and 79.1 PMP, respectively [31]. In 2008, 45,423 new patients started dialysis, representing an incidence rate of 36 PMP [31].

In 2010, Professor Chen Xiangmei (陈香美) established a national dialysis system called ‘Chinese National Renal Data System’(CNRDS), which is currently used [32,33]. Data from CNRDS revealed an increase in the numbers of prevalent PD patients annually from 2012 through 2017: 37942, 46633, 55373, 62589, 74138 and 86344, respectively. The corresponding point prevalence rates were 28.0, 34.3, 40.5, 45.6, 53.6, and 62.1 PMP, respectively. The numbers of new PD patients from 2012 to 2017 were 6930, 8691, 8740, 7216, 11549 and 12206, respectively; representing annual incidence rates of PD being 5.1, 6.3, 6.3, 5.2, 8.3 and 8.7 PMP, respectively [25]. In 2019 the number of PD patients had increased to 103,348 as reported by CNRDS at the annual conference of the Chinese Society of Nephrology in October 2020 (Figure 3). The number of new PD patients in 2019 had increased to 17,380. Meanwhile, the number of prevalent HD patients had an increase to 579,381 in 2018 and 632,653 in 2019, respectively. The numbers of new HD patients were 124,858 in 2018 and 134,640 in 2019, respectively (unpublished data). Although there has been a rapid rise in number of PD patients, this modality still lags behind the use of HD; in 2019 there were 632,653 HD patients and as discussed above 103,348 PD patients. It is noteworthy, though, that relatively more dialysis patients are using PD in China than anywhere else in the world. In 2014 it was estimated that 11% of all dialysis patients worldwide were on PD while for China this number was well over 35% [34].

Two major factors may limit the use of PD in China. First, the reimbursement policies have favored HD rather than PD in China. Second, patient management is centralized in tertiary care facilities and patients are cared by organized protocols and professionals; in contrast, PD patients at home are not as closely followed generating lower degree of sense of security and confidence. However, the recent implementation of chronic disease management in PD should improve the quality of care and confidence in this form of RRT [35].

Meanwhile, there has been an obvious increase in the number of PD centers nationwide. Data in 2002 showed that there were 347 PD centers in China, including 4 large (100-200 patients per center) PD centers; the rest of the PD centers were small-sized with less than 25 patients per center [36]. By December 31, 2017 data from CNRDS showed that there were 983 PD centers nationwide. Guangdong, Zhejiang, Jiangsu, Hunan and Liaoning ranked in the top five in China. In 2017 there were 106 PD centers and 12,236 PD patients in Guangdong Province, ranking the first in China [25].

## Clinical management of PD

In 2002 Professor Wang Tao (汪涛), director of PD center in Peking University First Hospital, established an innovative chronic disease management model. He pointed out ‘an effective management of chronic disease should be patient-centered.’ A team consisting of patients, professional nurses responsible for patients, nephrologists and a dietitian is involved in the management and follow-up of patients [37, 38]. This disease management model improved PD care significantly and led to the concept of ‘Center of Excellency’. Today, this model has been implemented nationwide.

Professor Wang and his team extended this model approach to the entire country. In July 2008, with the support of Baxter, the international kidney disease clinical research and training center (Baxter Sciencia) was established and guided by Professor Wang. This center devoted itself to training large number of medical professionals in the Asia Pacific region in the field of PD and chronic kidney disease (CKD). From 2008 to 2012 they held more than 50 international workshops in Beijing which were attended by many scholars from China, Hong Kong, Taiwan, Japan and Singapore.

In 2008, in view of the wide variation in the use of PD in various regions of China, Professor Yu Xueqing (余学清) in The First Affiliated Hospital of Sun Yat-sen University pointed out ‘PD management should be standardized in China’ and established the concept of ‘PD satellite centers’ with teams consisting of the central hospital and satellite PD centers. In the same year 13 PD centers in Guangzhou Province became satellite centers of The First Affiliated Hospital of Sun Yat-sen University. PD physicians and nurses in satellite centers came to the central hospital to receive professional training for 3-6 months. The establishment and successful practice of ‘PD satellite centers’ effectively promoted the development of PD in China and was known as the ‘Guangzhou model’ [39].

Afterwards in 2010, a Blood Purification Standard Operating Procedure (SOP) was promulgated to standardize dialysis management [9]. This required that dialysis complications and key quality indicators, such as hemoglobin, serum albumin, and dialysis adequacy, must be evaluated regularly.

## Clinical outcomes and quality indicators

It is noteworthy that there was a decline in the annual mortality rate of PD patients from 2012 to 2017: 5.7%, 4.3%, 3.0%, 2.8%, 2.9% and 2.3%, respectively (annual mortality rate = number of patients deaths/prevalent PD patients at the same year) [25]. The causes of death in peritoneal dialysis patients were similar to those in hemodialysis patients. Cardiovascular disease was the leading cause, accounting for 37.5% of deaths. Cerebrovascular accidents and infection were the second and third cause, accounting for 18.4% and 11.3% of deaths, respectively [25, 30].

Control of peritonitis is one of the key quality indicators in the management of PD programs. From 2012 to 2017 the incidence of PD-related peritonitis (total patient months/times) was 28.7, 26.3, 26.1, 29.1, 35.8 and 36.0, respectively [25].

According to the data from CNRDS, the percentage of PD patients with a hemoglobin level ≥10 g/dl from 2012 to 2017 was 38.9%, 42.8%, 44.7%, 52.2%, 53.2% and 57.8%, respectively. From 2012 to 2017, the percentage of PD patients with a serum albumin level > 35 g/l was 50.9%, 53.0%, 53.5%, 55.1%, 56.0% and 63.0% [25]. In 2017 49.3% of PD patients had a blood pressure level ≤140/90 mmHg, significant improvement from 19.6% in 2012 [24,25].

On the other hand, improvement has been challenging in other areas. In 2017 about 50.0% PD patients had blood phosphate within 1.13-1.78 mmol/l, which was similar to that of 2012. 55.6% PD patients in 2017 had intact parathyroid hormone (iPTH) within 150-600 pg/ml, which was a little higher than 52.7% in 2012. Furthermore 54.0% PD patients in 2017 had a Kt/V level ≥1.7, a little lower than 55.7% reported in 2012 [24,25].

## Current research and publications

Professor Yu from The First Affiliated Hospital of Sun Yat-sen University published a comprehensive proposal to ensure the success of PD. They emphasized the importance of CKD education, favorable reimbursement policy, committed physicians and nurses who are trained in the principles and practice of PD, the proper PD catheter insertion, PD patient training, appropriate regular follow-up, continuous quality improvement (CQI) and ongoing clinical research in the development of PD program [35].

A randomized control trial (RCT) in 2017 showed that CAPD regimens with 3 and 4 exchanges had similar effects on residual renal function, patient survival and technique survival [40]. Incremental peritoneal dialysis has been shown to be safe when patients are closely monitored [40,41]. Su *et al.* showed that lower dialysis dose (2 bags, 4,000mL/day) with lower daily protein intake can achieve a lower-level nitrogen balance and did not lead to malnutrition, indicating the importance of applying concept of nitrogen balance in assessing dialysis adequacy in this population. This approach may help those patients with poor economy to maintain adequate dialysis and survival [42].

Meanwhile, more interest is focused on intelligent PD patient care. Dr. Tang and colleagues used deep learning models to predict adverse outcomes in PD patients in their center in 2019 and showed that the recurrent neural network model, especially the gated recurrent unit (GRU) model, was more effective in predicting PD patient outcome as compared to the logistic regression (LR) model. This new model may help nephrologists to provide timely intervention and thus improve PD patient care [43].

A lot of interest has also been focused on PD catheter insertion in recent years. Percutaneous catheter placement either using modified Seldinger technique or ultrasound-guided technique has been successfully implemented in China and this has helped to promote the overall growth of PD in China [44].

With the increase of PD experience, the improvement of patient outcome and the use of new information technology (IT), we believe that PD activities in China may help to improve PD patient care worldwide.

### Future prospects

The growth in peritoneal dialysis in China in the past 60 years has been very substantial. We believe that, with the increase of PD experience, improved patient care particularly with the implementation of chronic disease management and quality care, the innovative payment regimens that favors PD rather than HD, and improved domestic PD products, PD use in China will increase rapidly in the coming years.

To further improve the use of PD, we suggest that more efforts should be focused on 1) the promotion of PD care in the rural areas where PD is still very limited due to the lack of PD awareness and education; 2) PD quality management and CQI program particularly focusing on PD adequacy and patient rehabilitation; 3) development and enforcement of national standards on PD management; 4) multi-center studies to compare the benefits of PD and HD including survival, rehabilitation and cost-effectiveness.

## References

[1] Wang ZG. Blood purification. 2nd edn. Beijing: Beijing Science and Technology Publishing House; 2003.

[2] Zhang SX, Ye RG, Li HQ, et al. Clinical study of peritoneal dialysis: 13 years of experience. Acad J Sun Yat-Sen Univ Med Sci. 1993;14(2):81–86.

[3] Zhao ZG. Clinical application of peritoneal dialysis: a report of 14 cases. Tianjin Yi Yao. 1975;4:196–201.

[4] Gu FL, Zhang JL. Peritoneal dialysis. Journal of Beijing Medical College. 1975;2:113–117.

[5] Ye RG, Li HQ, Yu Y. Rescue of patients with acute renal failure by peritoneal dialysis. Chinese J Crit Care Med. 1983;3(3):1–4.

[6] Ye RG, Li HQ, Zhang SG, et al. Long-term peritoneal dialysis for chronic renal failure: literature review and a report of 41 cases. Guangdong Med J. 1981;5:1–4.

[7] Ye RG. Modern peritoneal dialysis and its clinical application. Element Med. 1984;11:2–5.

[8] Ye RG. Renal failure and peritoneal dialysis. Guangdong: Guangdong Science and Technology Press; 1982.

[9] Ministry of Health of the People’s Republic of China. Blood purification standard operating procedure (SOP). Beijing: People’s Military Medical Press; 2010.

[10] Tenckhoff H, Schechter H. A bacteriologically safe peritoneal access device. Trans Am Soc Artif Intern Organs. 1968;14:181–187. 5701529

[11] Oreopoulos DG, Thodis E. The history of peritoneal dialysis: early years at toronto Western hospital. Dial Transplant. 2010;39(8):338–342.

[12] Ye RG, Li HQ, Xu NG. Experience and lessons of tenckhoff catheter implantation in peritoneal dialysis patients. New Medicine. 1981;12:176–178.

[13] Huang CY, Yao Q, Qian JQ. Icodextrin dialysate and peritoneal dialysis. J Shanghai Jiaotong University. 2009;29(4):470–473.

[14] Zhu TY, Hao CM. Clinical use of peritoneal dialysate. Chin J Prac Inter Med. 2013;33(6):442–444.

[15] Zhou JH, Ni ZH, Mei CL, et al. Efficacy and safety of changfu peritoneal dialysis solution: a multi-center prospective randomized controlled trial. Chin Med J. 2013;126(22):4204–4209. 24238498

[16] Li HQ, Ye RG, Xu NG, et al. Preliminary evaluation of dialysis device in peritoneal dialysis. J Nephrol Dialy Transplant. 1997; 6:151–152.

[17] Wang T, Zeng H, Ye RG, et al. Application of O-tube group for peritoneal dialysis. J Nephrol Dialy Transplant. 1993; 2(6):472–474.

[18] Lu Y. Comparison of clinical application of three kinds of devices in peritoneal dialysis. Shandong Med J. 2008;48(31):29.

[19] Popovich RP, Moncrief JW, Nolph KD, et al. Continuous ambulatory peritoneal dialysis. Ann Intern Med. 1978;88(4):449–456. 637423 10.7326/0003-4819-88-4-449

[20] Moncrief JW. The birth and development of continuous ambulatory peritoneal dialysis. Contrib Nephrol. 2017;189:85–90. 27951554 10.1159/000450689

[21] Li HQ, Ye RG, Yu Y, et al. A summary of continuous ambulatory peritoneal dialysis for 10 years of experience. Acad J Sun Yat Sen Univ Med Sci. 1992;13(2):65–69.

[22] Yu XQ, Yang X. Current situation and prospect of peritoneal dialysis in China. Chin J Kidney Dis Invest. 2012;1(1):12–15.

[23] Ni LS. The past, present and future of continuous ambulatory peritoneal dialysis. J Nephrol Dialy Transplant. 1994; 3(6):447–450.

[24] Dialysis and Transplantation Registration Working Group of Chinese Medical Association Kidney Disease Branch. A report of national dialysis transplantation registration in 1999. Chin J Kid Dis. 2001;17(2):77–78.

[25] Ministry of Health of the People’s Republic of China. National report on the services, quality and safety in medical treatment. Beijing: Beijing Science and Technology Publishing House; 2018.

[26] Lasker N, Shalhoub R, Habibe O, et al. The management of end-stage KIDNEY DISEASE WITH INTERMITTENT PERITONEAL DIALYSIS. Ann Intern Med. 1965; 62:1147–1169. 14295499 10.7326/0003-4819-62-6-1147

[27] Li ZH, Jin HJ. New application of automated peritoneal dialysis. Chin J Kid Dis Invest. 2015;4(1):10–13.

[28] Dong J, Zuo L. Are we ready to choose automated peritoneal dialysis? Chin J Nephrol. 2010;26(7):493–496.

[29] Ding XQ, Ni ZH, Jiang GR, et al. 60-years’ development of nephrology and dialysis technology in shanghai. Shanghai Med. 2017;40(1):8–13.

[30] Bi S-H, Mu B, Tang Z, et al. The history of hemodialysis in China. Hemodial Int. 2020;24(3):269–275. 31887231 10.1111/hdi.12815

[31] Zuo L, Wang M. Chinese association of blood purification management of chinese hospital association. Current burden and probable increasing incidence of ESRD in China. Clin Nephrol Suppl. 2010;74:S20–S22. 20979958

[32] Zhang LX, Zuo L. Current burden of end-stage kidney disease and its future trend in China. CN. 2016;86(S1):27–S28. 10.5414/CNP86S104 27469147

[33] Group of hemodialysis adequacy in nephrology branch, Chinese Medical Association. Clinical guideline of hemodialysis adequacy in China. Chin Med J. 2015;95(34):2748–2753.

[34] Li PK, Chow KM, Luijtgaarden V, et al. Changes in the worldwide epidemiology of peritoneal dialysis. Nat Rev Nephrol. 2017;13(2):90–103. 28029154 10.1038/nrneph.2016.181

[35] Yu XQ, Mehrotra R, Yang X. Components of a successful peritoneal dialysis program. Semin Nephrol. 2017;37(1):10–16. 28153189 10.1016/j.semnephrol.2016.10.003

[36] Yu XQ. Current situation and thinking of peritoneal dialysis in China. Chin J Integr Trad West Nephrol. 2005;6(1):1–2.

[37] Su CY, Lu XH, Chen W, et al. Promoting self-management improves the health status of patients having peritoneal dialysis. J Adv Nurs. 2009;65(7):1381–1389. 19457010 10.1111/j.1365-2648.2009.04993.x

[38] Cheng LT, Tang W, Wang T. Strong association between volume status and nutritional status in peritoneal dialysis patients. Am J Kidney Dis. 2005;45(5):891–902. 15861355 10.1053/j.ajkd.2005.01.037

[39] Yu XQ. The evolving patterns of uremia: unmet clinical needs in dialysis. Contrib Nephrol. 2017;191:1–7. 28910786 10.1159/000479251

[40] Yan H, Fang W, Lin A, et al. Three versus 4 daily exchanges and residual kidney function decline in incident CAPD patients: a randomized controlled trial. Am J Kidney Dis. 2017;69(4):506–513. 27751610 10.1053/j.ajkd.2016.08.019

[41] Liu XH, Dong J. Incremental peritoneal dialysis. Chin J Nephrol. 2020;36(3):253–256.

[42] Su CY, Wang T, Lu XH, et al. Low-dose dialysis combined with low protein intake can maintain nitrogen balance in peritoneal dialysis patients in poor economies. CN. 2017;87(02):84–92. 10.5414/CN108960 28074773

[43] Tang W, Gao JY, Ma XY, et al. Application of recurrent neural network in prognosis of peritoneal dialysis. J Peking Univ. 2019;51(3):602–610. 10.19723/j.issn.1671-167X.2019.03.034 PMC7439044 31209438

[44] Cheng SQ, Zhou TT, Zhang ZH, et al. Clinical application of ultrasound-guided seldinger percutaneous peritoneal dialysis catheterization. J Kid Dis Dialysis Renal Transplant. 2020;29(5):433–437.

